# Insulin-Like Growth Factor 1 Receptor Is a Prognostic Factor in Classical Hodgkin Lymphoma

**DOI:** 10.1371/journal.pone.0087474

**Published:** 2014-01-28

**Authors:** Zheng Liang, Arjan Diepstra, Chuanhui Xu, Gustaaf van Imhoff, Wouter Plattel, Anke Van Den Berg, Lydia Visser

**Affiliations:** 1 Department of Pathology and Medical Biology, University of Groningen and University Medical Center Groningen, Groningen, Netherlands; 2 Laboratory of Cancer Cell Biology, Tianjin Medical University Cancer Institute and Hospital, Tianjin, China; 3 Department of Otolaryngology, Tianjin Medical University General Hospital, Tianjin, China; 4 Department of Rheumatology and Immunology, Peking University People's Hospital, Beijing, China; 5 Department of Hematology, University of Groningen and University Medical Center Groningen, Groningen, Netherlands; University of Birmingham, United Kingdom

## Abstract

The interaction between the tumor cells in classical Hodgkin lymphoma (cHL) and the microenvironment includes aberrant activity of receptor tyrosine kinases. In this study we evaluated the expression, functionality and prognostic significance of Insulin-like growth factor-1 receptor (IGF-1R) in cHL. IGF-1R was overexpressed in 55% (44/80) of cHL patients. Phosphorylated IGF-1R was detectable in a minority of the IGF-1R positive tumor cells. The overall survival (OS, 98%) and 5-year progression-free survival (PFS, 93%) was significantly higher in IGF-1R positive cHL patients compared to IGF-1R negative patients (OS 83%, p = .029 and PFS 77%, p = .047, respectively). Three cHL cell lines showed expression of IGF-1R, with strong staining especially in the mitotic cells and expression of IGF-1. IGF-1 treatment had a prominent effect on the cell growth of L428 and L1236 cells and resulted in an increased phosphorylation of IGF1R, Akt and ERK. Inhibition of IGF-1R with cyclolignan picropodophyllin (PPP) decreased cell growth and induced a G2/M cell cycle arrest in all three cell lines. Moreover, a decrease in pCcd2 and an increase in CyclinB1 levels were observed which is consistent with the G2/M cell cycle arrest. In conclusion, IGF-1R expression in HRS cells predicts a favorable outcome, despite the oncogenic effect of IGF-1R in cHL cell lines.

## Introduction

Classical Hodgkin lymphoma (cHL) is characterized by a minority of malignant Hodgkin and Reed-Sternberg (HRS) cells that usually represent only about 1% of the total number of cells in the tumor tissue. The HRS cells are surrounded by a vast majority of reactive cells including lymphocytes, plasma cells, eosinophils and histiocytes [1].

HRS cells are dependent on interactions with other cell types for their survival. These interactions include, among others, tumor cell activation by multiple receptor tyrosine kinases (RTK), which have been shown to be overexpressed in HRS cells [2].

The Insulin-like Growth Factor 1 Receptor (IGF-1R) is a tetrameric receptor tyrosine kinase consisting of two ligand-binding extracellular α-subunits that are bound by disulfides to two single transmembrane β-subunits [3]. The molecular structure of its ligand Insulin-like Growth Factor 1 (IGF-1) is similar to Insulin. IGF-1 is produced primarily by the liver and bone marrow stromal cells as an endocrine factor, under the control of hypothalamic growth hormone releasing hormone and pituitary growth hormone.

A unique feature of IGF-1R, different from other RTKs, is that it is in a constitutive dimerized state, even in the absence of its ligand [4]–[6]. Upon ligand binding, the three tyrosine residues (Y1135, Y1131 and Y1136) are transphosphorylated by the tyrosine kinase (TK) domain of the β-subunit [7], resulting in an increase in catalytic activity. The phosphorylated tyrosine residues serve as docking sites for other signaling molecules such as insulin receptor substrate 1–4 (IRS-1-4) and SRC homology 2 domain-containing proteins (Shc). These molecules respectively activate the phosphoinositide 3 kinase (PI3K)/Akt and the mitogen-activated protein kinase (MAPK) pathways [3], [8], [9].

Another unique feature of IGF-1R is that at least three PI3K molecules can be recruited by one IGF-1R. PI3K binds directly to the pY1316 residue of the C-terminal domain of IGF-1R [10], and two additional PI3K molecules bind to pY608 and pY939 of IRS-1 [11]. Activation of Akt exerts anti-apoptotic effects through inhibitory phosphorylation of pro-apoptotic factors as BAD, as well as increased expression of anti-apoptotic proteins such as BCL-2, and BCL-XL [12].

PI3K was found to be constitutively activated in HRS cells and promoted their survival [13]. The MAPK pathway mediates diverse biological functions depending upon the cellular context, including cell growth, survival, and differentiation [14]. Aberrant IGF-1 signaling has been found in multiple aspects of tumor biology, including proliferation, transformation, apoptosis protection and chemotherapy-resistance [15]–[17]. In hematopoietic malignancies, a critical role was shown of the IGF-1/IGF-1R signaling pathway for proliferation and survival in multiple myeloma (MM) [18] and mantle cell lymphoma (MCL) [19]. The functionality of IGF-1R in cHL is unknown. In this study we evaluated the expression, function and prognostic significance of IGF-1R in cHL.

## Materials and Methods

### Patient and tissue data

Primary cHL tissues were retrieved from the Department of Pathology, University Medical Center Groningen, the Netherlands (n = 80 collected from 1993 to 2010). The basic characteristics of the patients are presented in Table 1. The histological diagnosis was based on the currently used criteria defined by the World Health Organization 2008 classification. The median follow-up was 55 months (interquartile range, 34.5–104.5 months). The study protocol was consistent with international ethical guidelines (the Declaration of Helsinki and the International Conference on Harmonization Guidelines for Good Clinical Practice). The same patient cohort was used in an earlier study [20], and according to the Medical ethics review board of the University Medical Center Groningen fulfilled requirements for patient anonymity and were in accordance with their regulations. The Medical ethics review board waives the need for approval if rest material is used under law in the Netherlands, and waives the need for informed consent when patient anonymity is assured.

**Table 1:** IGF-1R expression by HRS cells in relation to disease parameters, EBV status and treatment.

Characteristic	IGF1R+ (n = 44, 55%)	IGF1R- (n = 36, 45%)	p-value
**Median age (range)**	25 (7–83)	35 (8–69)	0.14*
**Gender**			0.53†
- male	24 (55%)	19 (53%)	
- female	20 (45%)	17 (47%)	
**Histology subtype**			0.02†
- NS	38 (86%)	23 (64%)	
- MC	1 (2%)	8 (22%)	
- LR	1 (2%)	-	
- cHL, NOS	4 (9%)	5 (14%)	
**Ann Arbor Stage**			0.93†
- I-II	27 (61%)	21 (58%)	
- III-IV	15 (34%)	14 (39%)	
- n.a.	2 (5%)	1 (3%)	
**EBV status**			0.36†
- positive	11 (25%)	12 (33%)	
- negative	33 (75%)	23 (64%)	
- n.a.	-	1 (3%)	
**B-symptoms**			0.83†
-present	25 (57%)	19 (53%)	
-absent	17 (39%)	16 (44%)	
-n.a.	2 (5%)	1 (3%)	
**IPS**			0.67†
-low (0-2)	28 (64%)	22 (61%)	
-high (≥3)	9 (21%)	10 (28%)	
-n.a.	7 (16%)	4 (11%)	
**Chemotherapy**			0.71†
-ABVD	20 (45%)	22 (61%)	
-BEACOPP	1 (2%)	1 (3%)	
-MOPP/ABVD	7 (16%)	5(14%)	
-Pediatric protocols	12 (27%)	6 (17%)	
-RT alone	2 (5%)	1 (3%)	
-n.a.	2 (5%)	1 (3%)	

NS: nodular sclerosis; MC: mixed cellularity; LR: lymphocyte rich; LD: lymphocyte depleted; NOS: not otherwise specified; n.a.  =  not available; *Mann-Whitney test; †Pearson's chi-squared test.

### Immunohistochemistry

Formalin-fixed and paraffin-embedded specimens were cut into 4-µm sections, mounted onto polylysine-coated slides, deparaffinized in xylene, and rehydrated in a graded ethanol series. The sections were quenched for endogenous peroxidase with 0.3% hydrogen peroxide, and then boiled in EDTA (1 mmol/L; pH8.0) for 15 minutes in a microwave oven for antigen retrieval. The sections were incubated at 37°C for 1 hour with the first antibody, IGF-1R (1∶1000, (3C8B1, 3G5C1 [21], [22]), Abcam, Cambridge, UK) pIGF-1R (Y1161, Abcam) and CD30 (BerH2, Dako, Glostrup, Denmark), and 30 minutes with horse-radish peroxidase-labeled secondary and tertiary antibodies (Dako). 3,3′-diaminobenzidine (DAB) chromogen (Sigma Aldrich, St Louis, MO, USA) was used as substrate and sections were counterstained with hematoxylin. CD30 expression was used to identify the tumor cells. For positive controls tonsil and breast carcinoma tissue was used. For negative controls, tissue sections were incubated under the same experimental conditions without primary antibody. For immunocytochemistry on the 3 cHL cell lines, cytocentrifuge preparations were made and fixed with acetone for 10 minutes. Cells were stained with the primary antibody, and subsequently incubated with secondary and third antibodies; 3-Amino-9-ethylcarbazole was used as a chromogen.

Evaluation of IGF-1R expression was performed by a hematopathologist without knowledge of the clinical outcome. A case was defined positive when HRS cells showed homogenous staining for IGF-1R. Blood vessels in all tissue sections served as internal controls for IGF-1R. Expression of IGF-1R was correlated to age, sex, histological subtype, stage of disease, EBV status and with other known prognostic factors.

### Cell lines

The cHL cell lines L428, KM-H2 and L1236 (DSMZ, Braunschweig, Germany) were cultured in RPMI-1640 medium supplemented with10% fetal calf serum (5% for L428), ultraglutamine, and 100 U/ml penicillin/streptomycin (Lonza, Verviers, Belgium).

### IGF-1 levels in cHL cell line culture supernatant

2*10^5^ cells per ml were cultured in triplicate in RPMI 1640 with FCS and supernatants were collected after 24 hours. IGF-1 protein levels were measured by ELISA (R&D Systems, Minneapolis, MN, USA) according to the instructions provided by the manufacturer. RPMI 1640 supplemented with 10% FCS was used as the baseline for the levels of secreted IGF-1.

### Western blot analysis

Cell lines were starved in medium without serum for 24 hours and treated with different concentrations of an IGF-1R inhibitor *cyclolignan picropodophyllin* (PPP; Calbiochem, Gibbstown, NJ, USA)) for 4 hours. PPP stock solutions were prepared in DMSO (Sigma-Aldrich) and stored at 4°C in 10 uM aliquots. After 30 minutes of incubation with exogenous IGF-1 (R&D Systems) at a concentration of 50 ng/mL, cells were lysed in lysis buffer (Cell Signalling Technology, Boston, MA, USA) and incubated for 20 minutes on ice. The cell lysate was centrifuged at 14,000 g for 10 minutes at 4°C to remove cellular debris. The protein concentration was determined using bicinchonic acid (Pierce Chemical Co., Rockford, IL, USA) according to the manufacturer's instructions. The cell lysate was diluted in loading buffer, boiled for 5 minutes and separated on a 8% or 12% (w/v) Tris-HCl SDS polyacrylamide gel. Proteins were transferred to a nitrocellulose membrane using standard protocols and incubated with primary antibodies at 4°C overnight. Immunostaining was amplified by incubation with HRP-conjugated antibodies (Dako) and chemiluminescence was detected using SuperSignal®West Dura Extended Duration Substrate (Pierce Chemical Co). Experiments were performed in triplicate. Antibodies used in the Western blot analyses were anti-IGF-1R, anti-pIGF-1R (Tyr1135/1136), anti-Akt, anti-pAkt (D9E, Ser473), anti- 44/42 MAPK (ERK1/2), anti-p44/42 MAPK (ERK1/2, Thr202/Tyr204), anti-Cdc2, anti-pCdc2 (Tyr15), anti-CyclinB1 (V152)(Cell Signaling Technology) and β–actin (Abcam).

### Alamar blue test

Cellular growth after stimulation and inhibition was evaluated by alamar blue tests (AbDSerotec, Oxford, UK). Alamar blue is a measurement of metabolic activity, which can be measured at several time points in the same cells. In the absence of apoptosis, we used alamar blue as a measurement of relative cell numbers. Cell lines were seeded at 10,000 cells/ml in 96-well plates and 10 µL of alamar blue was added to each well. The fluorescence was measured with an excitation of 560 nm and emission at 590 nm. Every cell line was analyzed in triplicate (3 wells) and in three independent experiments. The fluorescence was measured with increasing concentrations of IGF-1, PPP or DMSO control at different time points. Relative percentages of cell growth were calculated as follows: mean fluorescence treated cells/mean fluorescence control cells.

### Cell cycle assays

For the cell cycle analyses, cells were harvested after 24 hours, washed in phosphate-buffered saline with 0.1% bovine serum albumin, and resuspended in 0.5 ml hypotonic PI solution (0.1% sodium citrate, 0.3% triton X-100, 0.01% propidium iodide and 0.002% ribonucease A). The percentage of cells in specific cell cycle phases (G1, S, and G2/M) was determined using a flow cytometer (Calibur, BD Biosciences, San Jose, CA USA) and the Modfit software. Every sample was tested in three independent experiments.

### Statistical evaluation

The IBM SPSS Statistics 20 software package and GraphPad Prism were used for the statistical analysis and data plotting. The Chi-square test or Fisher's exact test were used to analyze the associations between clinicopathological features with the IGF1R expressions, as appropriate. The nonparametric Mann-Whitney test was used to evaluate the association between patient age and IGF-1R expression. Proliferation and cell cycle results were analyzed by a student t-test or one-way analysis of variance (ANOVA).

Overall survival (OS) was measured from the date of diagnosis of cHL to the date of death. 5-years Progression-free survival (PFS) was defined as months elapsed between the date of diagnosis and the date of tumor progression or death from any cause, with censoring of patients who were lost to follow-up or still alive and free of cHL disease at the last date of follow-up. Survival curves were generated using the method of Kaplan-Meier. The log-rank test was used to evaluate statistical significance of the differences in the curves.

## Results

### IGF-1R expression in cHL tissue

**Figure 1:**
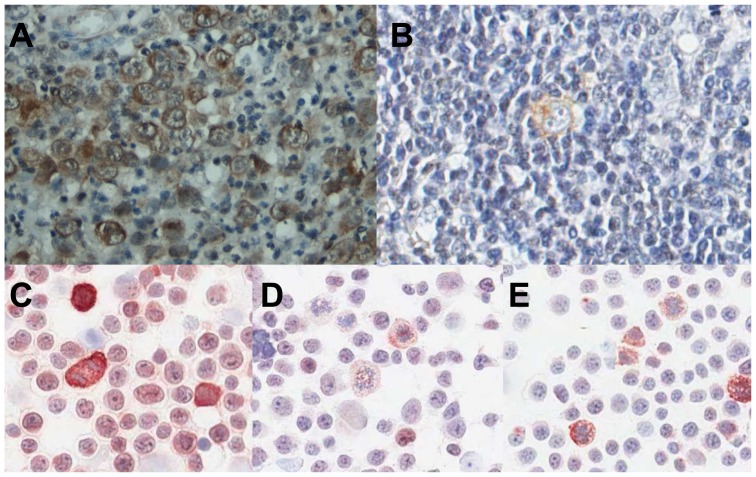
IGF-1R expression in HRS cells. A, Representative cHL case showing expression of IGF-1R in the vast majority of HRS cells. B, pIGF-1R positive HRS cell. C, IGF-1R expression in L428, showing positive staining in all cells with strong expression in mitotic cells, D, IGF-1R expression in L1236, showing weak staining in all cells and strong expression in mitotic cells and, E, IGF-1R expression in KM-H2, showing weak staining with more pronounced expression in mitotic cells.

The clinicopathological data of our patient cohort are summarized in Table 1. Expression of IGF-1R was cytoplasmic and/or membranous (Figure 1A) in 55% (44/80) of the cHL patients. Expression of IGF-1R was observed in all or the vast majority of HRS cells in positive cases, whereas in negative cases virtually all HRS cells were negative. There was a significant difference in IGF-1R expression with HL subtype (p = .0157), with a high percentage of positive cases in the nodular sclerosis cases (38/61) and a low percentage of positive cases in mixed cellularity cases (1/9). We found no statistical significant differences in IGF-1R expression for age, EBV status, Ann Arbor Stage, IPS, B-symptoms, bulky disease, or treatment. In a pilot study 16 IGF-1R positive patients were stained for pIGF-1R. Only single HRS cells stained positive for phosphorylated IGF-1R (Figure 1B) in 3 of the patients, whereas the HRS cells in the remaining 13 patients were pIGF-1R negative in all HRS cells. Based on this low percentage of positive cells we decided not to stain the whole patient cohort.

### Prognostic value of IGF-1R

**Figure 2:**
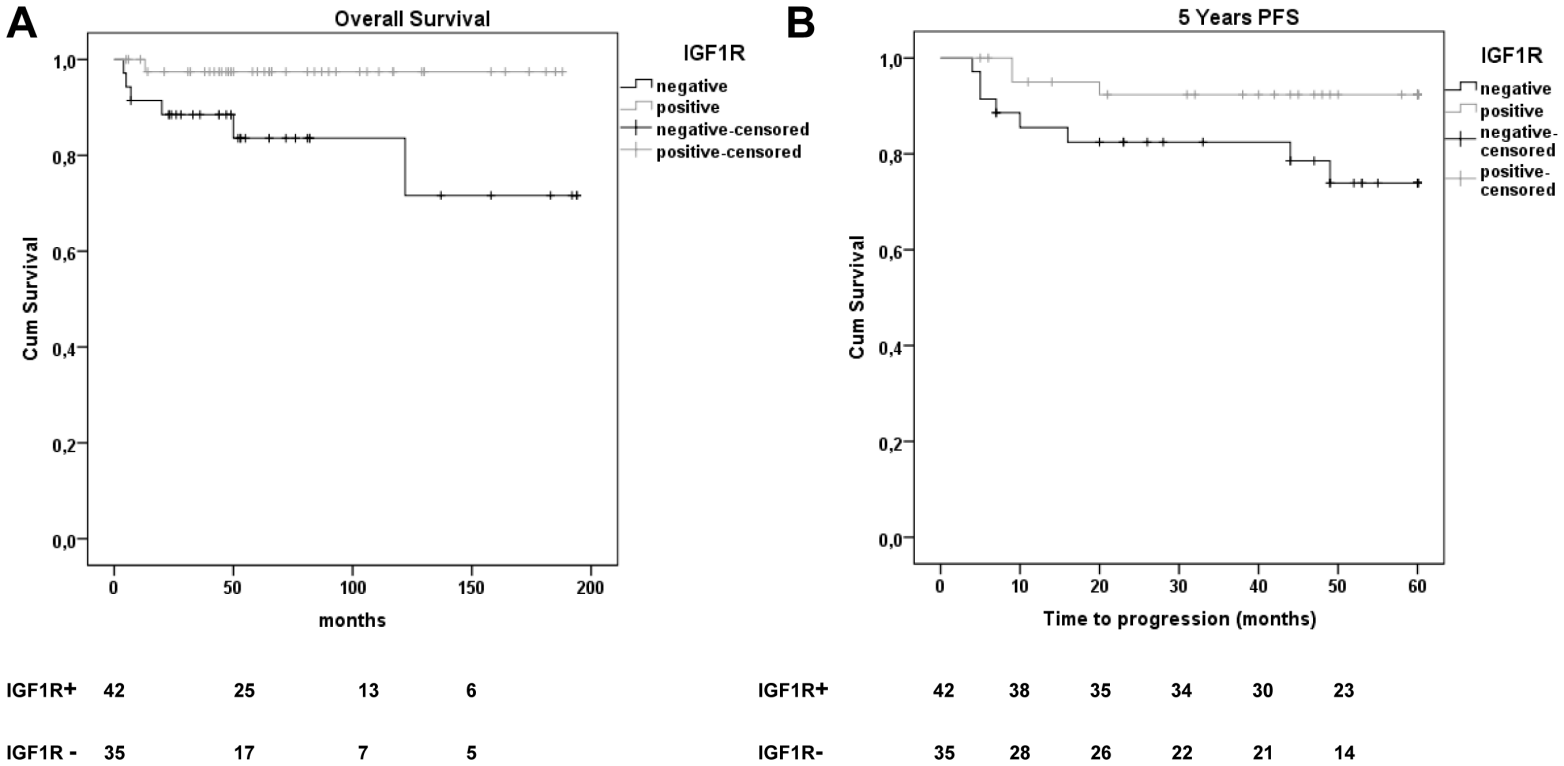
Kaplan-Meier curves for progression free survival (PFS) and overall survival (OS) in IGF-1R expression defined subgroups of cHL patients. The overall survival was lower in the IGF-1R negative group (83%) as compared to the IGF-1R positive group (98%; P = 0.029). The 5-year PFS was lower in the IGF-1R negative group (77%) as compared to the IGF-1R positive group (93%; P = 0.047). Numbers below the graph are the number of patients for each group included at that time point.

We studied the prognostic value of IGF-1R in our patient cohort. The overall survival (OS) was significantly higher in the IGF-1R positive patient group (98%), as compared to the IGF-1R negative patient group (83%, Figure 2A, p = .029). The 5-year progression-free survival (PFS) was also significantly better in IGF-1R positive patients (93%) compared to IGF-1R negative patients (77%, p = .047, Figure 2B). Thus, absence of IGF-1R expression is a significant prognostic factor for poor 5-years PFS in our cHL cohort. This prognostic value of IGF-1R was observed in the entire cohort, but in subgroup analysis according to stage only remained prognostic among advanced stage patients due to low number of cases and events per subgroup. The International Prognostic Score (IPS) could not predict prognosis among advanced stage patients in our cohort.

### IGF-1R and IGF-1 expression in three cHL cell lines

**Figure 3:**
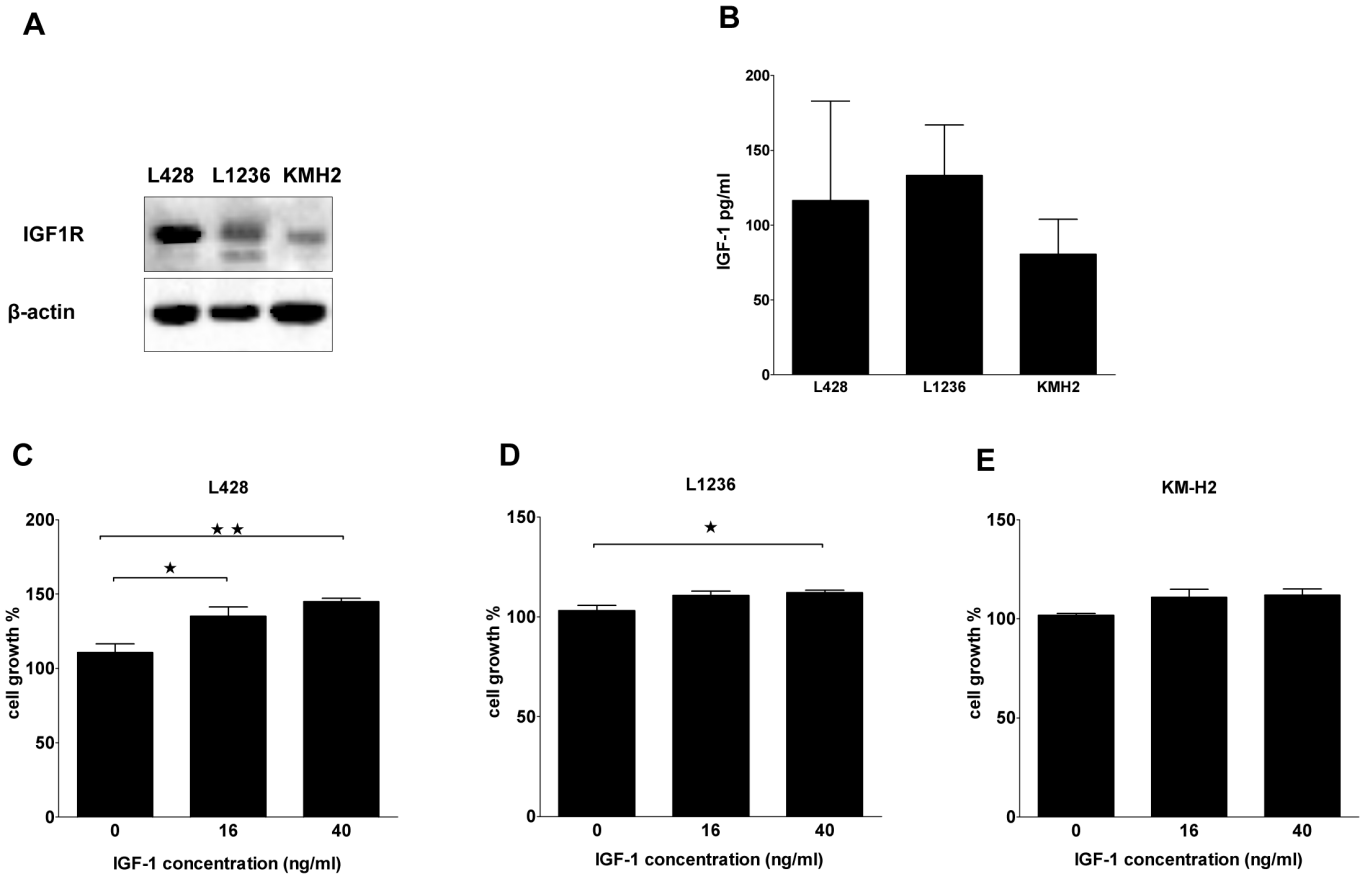
IGF-1R and IGF-1 expression and the effect of IGF-1 on growth in cHL cell lines. A, Western blot showing expression of IGF-1R in L428, KM-H2 and L1236. The IGF-1R level was high in L428, moderate in L1236 and low in KM-H2. B, IGF1 protein levels in the culture supernatant of cHL cell lines. The IGF-1 protein levels were similar in the three cHL cell lines. C, IGF-1 treatment of L428 cells revealed a significant effect on cell growth. D, L1236 cells showed a significant effect on cell growth upon IGF-1 treatment. E, no effect was seen in KM-H2 cells upon treatment with IGF-1.

The 3 cHL cell lines showed IGF-1R staining (Figure 1C, D, E), with the strongest expression in L428. In all 3 cell lines mitotic cells expressed higher levels of IGF-1R. Western blot analysis of the three cHL cell lines revealed different levels of IGF-1Rβ subunits (Figure 3A). L428 cells expressed the highest level of IGF-1R and KM-H2 the lowest. In order to evaluate a potential autocrine IGF-1R activation loop, we investigated the production of IGF-1 by the three cHL cell lines. The IGF-1 level was approximately 100 pg/ml in the cell culture supernatant after 24 hours of culture and similar levels were found for all three cell lines (Figure 3B).

### L428 cell growth is enhanced by IGF-1

In an attempt to evaluate the potential of IGF-1 stimulation, we incubated the three cell lines in medium with FCS with different concentrations of IGF-1. This revealed no impact on cell growth (data not shown), suggesting that the production of IGF-1 by the HL cells could be saturating the IGF-1R or that the production of other factors by the cells masks the putative effect of IGF-1. Next we performed the same experiment after overnight serum starvation. 72 hrs of IGF-1 treatment had the most prominent and significant effect (31% increase) on the cell growth of L428 cells (Figure 3C). In L1236 a significant increase in cell growth was observed only for the high dose of IGF-1 (40 ng/ml, Figure 3D). No significant effect was observed for KM-H2 (Figure 3E). Thus L428 cells were most sensitive to IGF-1 treatment, consistent with its high IGF-1R expression level.

### PPP inhibits cell growth of cHL cell lines in a dose and time dependant way

**Figure 4:**
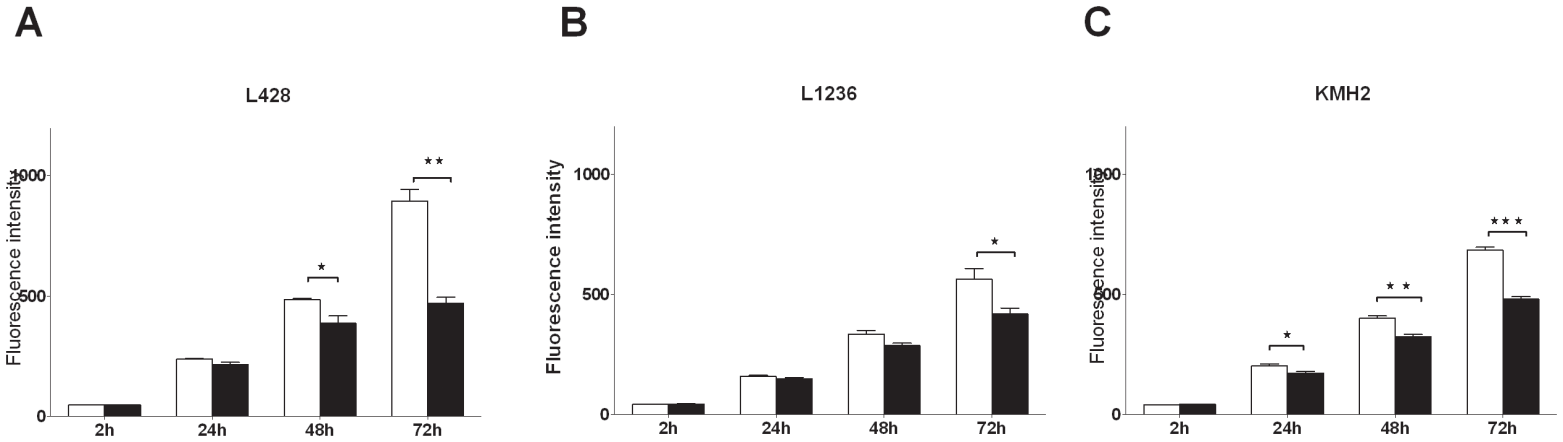
The effect of PPP on growth of cHL cell lines. A, L428 cells. B, L1236 cells. C, KM-H2 cells. Cell growth upon treatment with 2 µM PPP (black bars) for 1, 2 and 3 days was compared with untreated/DMSO control treatment (white bars). Cell growth was significantly decreased (*P*<0.001) in all three cell lines.

We next investigated the *in vitro* anti-proliferative effect of PPP (0.25–2 µM), without addition of IGF-1, in three cHL cell lines. PPP significantly suppressed cell growth in a dose dependent manner with the strongest effects observed at 1 and 2 µM PPP (Figure S1). The effect of PPP was time dependent and after 72 hours of PPP treatment the viability decreased significantly in all three cHL cell lines (Figure 4). The strongest effects were again observed for L428 cells with a reduction in cell growth of 48%. The reduction in cell growth was 30% in KM-H2 cells and 26% in L1236. The higher sensitivity of L428 for PPP is consistent with the high expression of IGF-1R on L428 cells and the sensitivity to IGF-1.

### PPP induces G2/M-phase arrest in cHL cell lines after 24 hours of PPP treatment

**Figure 5:**
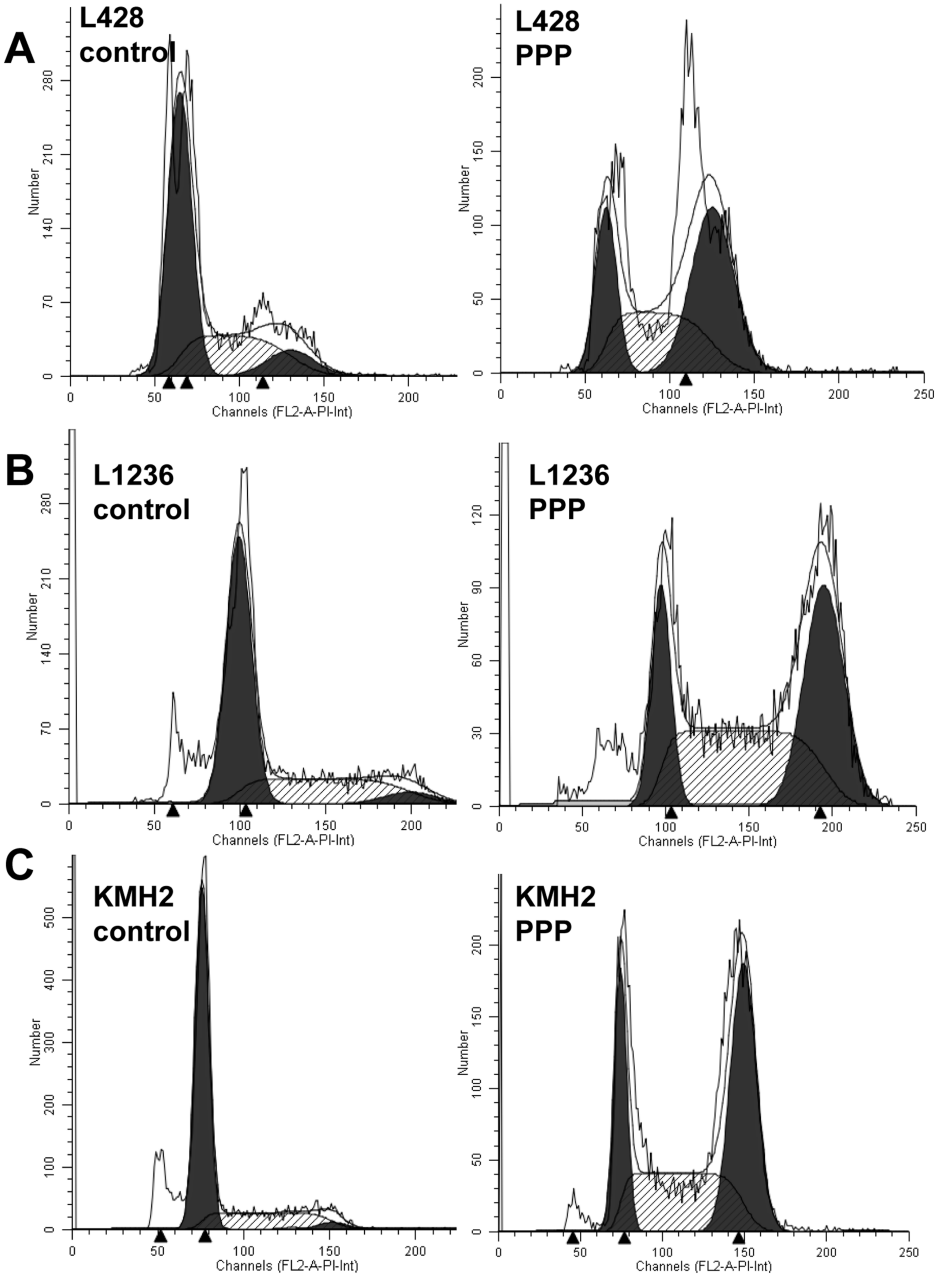
The effect of PPP on cell cycle progression of cHL cell lines. A, L428 cell cycle analysis. B, L1236 cell cycle analysis. C, KM-H2 cell cycle analysis. Flow cytometry histograms are given with a fitted analysis model, in which dark gray areas are calculated areas for cells in G0/G1 and G2 phase and the shaded area is the calculated area for cells in S phase. Cell cycle distribution upon treatment with PPP (2 µM)-treated for 24 hours revealed a clear G2/M cell cycle arrest in all cell lines. Representative figures are given. Results of triplicate experiments are given in supplementary figure 2.

To determine whether inhibition of IGF-1R by PPP has an effect on the cell cycle, we analyzed distribution of cells over the different cell cycle fractions by PI staining after 24 hours of treatment. Treatment with PPP induced G2/M cell cycle arrest in all three cell lines, the number of cells in the G2/M phase significantly increased from 8% to 45% in L428, from 4% to 48% in KM-H2 and from 3% to 38% in L1236 (Figure 5, and figure S2).

### The IGF-1R/IGF-1 signaling pathway in cHL cell lines

**Figure 6:**
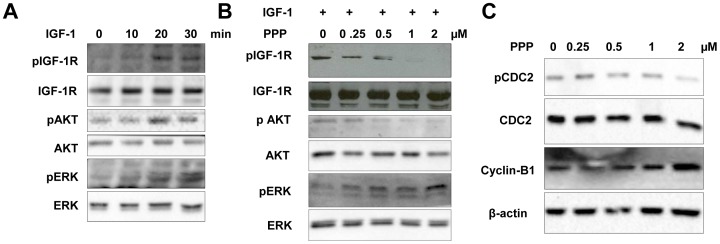
The effects of IGF-1 and PPP on IGF-1R autophosphorylation and on its downstream signalling proteins and cell cycle proteins in L428 cells. A, pIGF-1R level was measured at different time points after stimulation with IGF-1 (50 ng/mL). Phosphorylation levels of IGF-1R, Akt and ERK were increased upon IGF-1 stimulation at 20 and 30 minutes. B, phosphorylation of IGF-1R, and Akt upon IGF-1 stimulation could be blocked by the pIGF-1R kinase inhibitor PPP in L428 cells. While phosphorylation of ERK was increased by PPP (C) PPP treatment induced a reduction of Cdc2 phosphorylation and upregulation of CyclinB1, consistent with the occurrence of a G2/M-phase cell cycle arrest.

L428 has the highest expression of IGF-1R, so we used L428 for studying the IGF-1R/IGF-1 signaling pathway after serum starvation with IGF-1 stimulation and PPP inhibition. The optimal time to get a strong signal of IGF-1R phosphorylation by IGF-1 in L428 was 20–30 minutes (Figure 6A). Stimulation with IGF-1 revealed an increase of pAkt and pERK1/2 (Figure 6A). After treatment with PPP the IGF-1 induced phosphorylation of the IGF-1Rβ subunit is blocked, while the total amount of IGF-1Rβ was not affected. The induction of pAkt was effectively blocked by PPP in a dose dependent manner without altering Akt protein levels. Induction of ERK phosphorylation was induced by PPP (Figure 6B).

We next studied the effect of PPP on cell cycle regulators, including Cdc-2, pCdc-2 and CyclinB1. PPP inhibited phosphorylation of the cell cycle regulator Cdc-2 and increased CyclinB1, changes that are consistent with G2/M-phase cell cycle arrest (Figure 6C).

## Discussion

The pathogenesis of cHL remains poorly understood. HRS cells are derived from germinal center B cells that have lost their B cell phenotype including immunoglobulin expression [23]. Although normal germinal center B cells that lack functional immunoglobulin undergo apoptosis, HRS cells escape this programmed cell death [24], [25]. Aberrant activity of multiple RTKs in HRS cells might prevent apoptosis and explain resistance to treatment-induced apoptosis and treatment failure. HRS cells aberrantly express up to 7 different RTKs with extensive heterogeneity regarding the number and combinations in individual cases [2]. In general, a more prominent co-expression has been observed in nodular sclerosis than mixed cellularity cHL [26]. Consistent with these findings we also found that IGF-1R is predominantly expressed in the nodular sclerosis subtype of cHL, although we analyzed a limited number of mixed cellularity HL patients. Overall we demonstrated IGF-1R expression in the vast majority of the HRS cells in 55% of the cHL patients. IGF-1R is not normally expressed by B cells, only some large germinal center B cells show weak expression, but it is expressed by plasma cells [19]. IGF-1R is expressed by mantle cell lymphomas [19]. So, it is possible that expression of IGF-1R by the tumor cells in HL can be an effect of malignant transformation or due to differentiation towards plasma cells. In cHL expression of IRF4/Mum1, expressed in germinal center cells and plasma cells, and Blimp-1, a regulator of plasma cell differentiation, are both detected, although for Blimp-1 only in 23% of patients in a small part of tumor cells [27].

We studied expression of IGF-1R and IGF-1 in three cHL cell lines and observed marked differences in IGF-1R levels, whereas the levels of IGF-1 production were similar. Cells in the process of mitosis showed strongly enhanced expression of IGF-1R in all 3 cell lines. The functionality of the IGF-1R/IGF-1 signaling pathway was studied in L428 cells, which showed the highest IGF-1R levels. Consistent with its known signaling pathway we observed enhanced pIGF-1R, pAkt and pERK1/2 levels upon stimulation with IGF-1. Treatment with the IGF-1R inhibitor PPP revealed an inhibiting effect on cell growth, and blocked the constitutive phosphorylation of IGF-1R. It has been shown that the inhibitory effect of PPP on IGF-IR did not co-inhibit the insulin receptor activity or compete with ATP in *in vitro* kinase assays in several cell lines, suggesting that it may inhibit IGF-1R autophosphorylation at the substrate level [28]. Although PPP blocked phosphorylation of Akt, it induced phosphorylation of ERK1/2. These findings are similar to the results previously reported in melanoma cell lines [29]. PPP possibly induces ubiquitination of IGF-1R and in turn activates ERK1/2.

In all cell lines PPP caused G2/M cell cycle arrest. Blocking of the cell cycle is consistent with the decrease in pCdc2 (Tyr15) and increase in CyclinB1. In MCL cell lines PPP also induced a G2/M cell cycle arrest, with similar decreased pCdc2 and increased CyclinB1 levels. In the same study inhibition of IGF-1R with siRNA was also tested, since small molecule inhibitors can have nonspecific effects, and this resulted in very similar effects. In the MCL cell lines apoptosis was induced after 24 hours of treatment [19], while we did not see an increase of apoptotic cells between 4 to 72 hours of PPP treatment (data not shown). The PPP dependent effect on the G2/M cell cycle arrest correlates with the upregulation of IGF-1R expression during mitosis, and indicates the relevance of IGF-1R signaling for cell division. This might also fit the low frequency of pIGF-1R positive HRS cells and is consistent with the low number of HRS cells in mitosis in cHL tissue. The effect of PPP also highlights a potential therapeutic application of IGF-1R kinase inhibitors in combination with conventional G2 phase specific drugs (Vincristin and Bleomycin) included in the commonly used ABVD regimen in cHL.

Expression of IGF-1R has been shown to correlate with an unfavorable prognosis in several types of cancer, e.g. in advanced oral squamous cell carcinoma [30], but also with a favorable prognosis in studies in breast cancer [31]–[33], non small cell lung cancer [34] and soft tissue sarcoma [35]. In our cohort IGF-1R positivity was a significant predictor of good outcome in patients with cHL both for OS and 5-years PFS. How can the adverse prognosis of IGF-1R negative HRS cells be explained? Previous studies revealed that low IGF-1R expression is associated with de-differentiation of various human cancers[36]–[39], indicating that IGF-1R expression is lost when cells become more malignant. Increased MDM2 expression can cause downregulation of IGF-1R and in some cancers, such as soft tissue sarcoma, MDM2 expression is associated with a more malignant phenotype [40]. In cHL patients, expression of MDM2 is not associated with prognosis [41], and we did not find a correlation of MDM2 expression and absence of IGF-1R receptor expression in our patient cohort (data not shown). Another more likely explanation for this unexpected positive prognostic effect might be related to the characteristic histology of cHL with only a very small percentage of tumor cells in an abundant reactive infiltrate. The IGF-1/IGF-1R pathway plays important roles in hematopoietic cell growth and differentiation and normal immune function [42]. There is some evidence of a role for IGF-1R in T cell growth and function. Activated T cells have an increased number of IGF-1R molecules, and IGF-1 was shown to enhance anti-CD3 stimulated proliferation [43]. It can be speculated that activation of IGF-1R in HRS cells might alter the immunological response of the reactive cells by shaping the microenvironment in a way that is less beneficial for survival and growth of the HRS cells.

We recently reported that expression of the oncogenic RTK c-Met by HRS cells was a marker for good prognosis in HL [20]. Functional studies showed that the effect of c-Met inhibitors was unfavorable for growth of HL cell lines similar to the effects of IGF-1R inhibition. There was no correlation of c-Met expression with IGF-1R expression in this cohort (data not shown). In a recently published gene expression based model for the prediction of overall survival, low IGF-1 levels were correlated with a bad prognosis [44]. These findings are consistent with our results and support our findings that an active IGF-1/IGF-1R pathway is a favorable prognostic marker in HL.

In conclusion, we showed expression of IGF-1R in 55% of cHL patients. In cHL cell lines both IGF-1R and IGF-1 were expressed and inhibition of IGF-1R induced G2/M arrest. IGF-1R expression is enhanced in mitotic cells. Despite the oncogenic effect of IGF-1R in cell lines, IGF-1R expression in HRS cells predicts a favorable outcome and might be included in future clinical risk stratification after validation in future large prospective studies.

## Supporting Information

Figure S1 **Effect of PPP inhibition on cell growth.**
(DOC)

Figure S2 **Cell cycle distribution of PPP (2 µM) treated 3 cHL cell lines.**
(DOC)
