# Supplementary material for: Insulin-Like Growth Factor 1 Receptor Is a Prognostic Factor in Classical Hodgkin Lymphoma
Source: PLoS One. 2014 Jan 28;9(1):e87474. doi: 10.1371/journal.pone.0087474 (PMC3905016; doi:10.1371/journal.pone.0087474)

**Figure S1. Effect of PPP inhibition on cell growth.** 3 cHL cells were grown in the presence of increasing concentrations of PPP (0, 0.5, 1, 1.5, 2 μM) for 72 hours, and the effect on cell growth was assayed by Alamar Blue. Dose dependent effects of PPP were observed for all 3 cell lines.


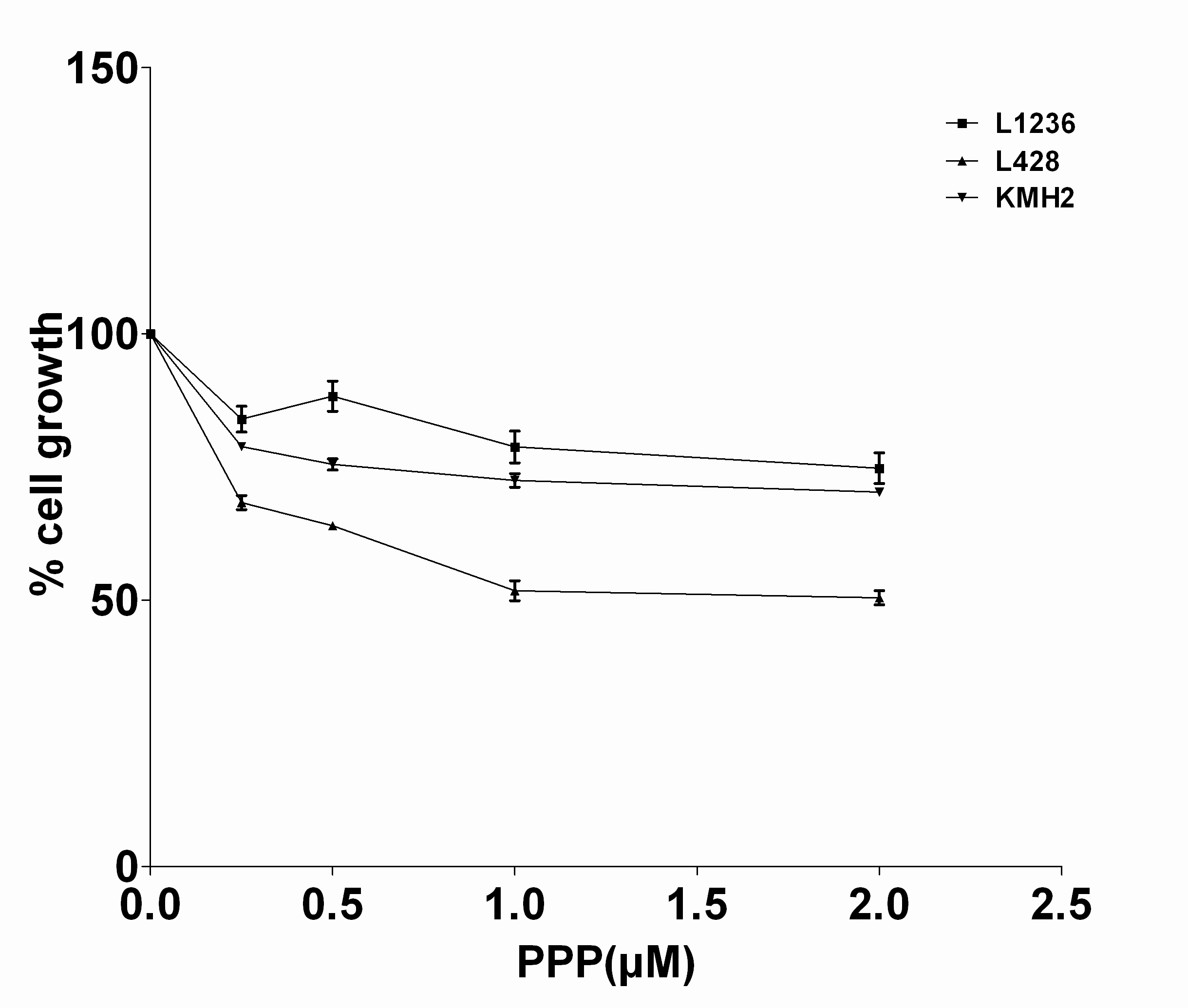

Supplement: Figure S1 — Effect of PPP inhibition on cell growth. (DOC) [file pone.0087474.s001.doc]
