# Supplementary material for: Insulin-Like Growth Factor 1 Receptor Is a Prognostic Factor in Classical Hodgkin Lymphoma
Source: PLoS One. 2014 Jan 28;9(1):e87474. doi: 10.1371/journal.pone.0087474 (PMC3905016; doi:10.1371/journal.pone.0087474)
Supplement: Figure S2 — Cell cycle distribution of PPP (2 µM) treated 3 cHL cell lines. (DOC) [file pone.0087474.s002.doc]

**Figure S2. Cell cycle distribution of PPP (2 μM) treated 3 cHL cell lines**. Flow cytometry histograms shows G2/M cell cycle arrest is an effect of IGF1R inhibition.
